# Supplementary material for: MetaRibo-Seq measures translation in microbiomes
Source: Nat Commun. 2020 Jun 29;11:3268. doi: 10.1038/s41467-020-17081-z (PMC7324362; doi:10.1038/s41467-020-17081-z)
Supplement: Supplementary file 10 — Supplementary Data 7 [file 41467_2020_17081_MOESM10_ESM.zip › File2/Confidence_High_Taxonomy/313557_out.krona.html]

Javascript must be enabled to view this page.

members
magnitude
magnitudeUnassigned
count
unassigned
taxon
rank

313557\_out

3

2
superkingdom
3

phylum
3
976

200643
3
class

171549
order
3

815
3
family

3
genus
816

1929999
species

SRS056273\_contig\_number\_21449SRS075078\_contig\_number\_26222SRS142599\_contig\_number\_19413
3
